# Supplementary material for: Developing and validating modular surveys for vector-borne diseases: A study protocol
Source: PLoS One. 2025 Aug 25;20(8):e0323072. doi: 10.1371/journal.pone.0323072 (PMC12377612; doi:10.1371/journal.pone.0323072)
Supplement: S1 Table — (DOCX) [file pone.0323072.s001.docx]

| Theme | ​​​Search Terms |
| --- | --- |
|  | “KAP” OR “KAB” OR “KAPP” AND    "west nile" OR "west nile virus OR dengue OR zika OR chikungunya OR malaria OR lyme OR "Rocky Mountain Spotted Fever" OR "Arbovirus Infections" OR Borrelia OR "Borrelia burgdorferi" OR "Powassan virus" OR "Rickettsiaceae Infections" OR "Tick-Borne Diseases" OR "saint louis ecephalitis" OR SLE OR "tickborne diseases" OR "mosquito-borne diseases" OR "vectorborne disease" OR VBD OR anaplasmosis OR "Powassan virus disease" OR ehrlichiosis OR babesiosis OR mosquitoborne OR vector-borne OR tick-borne OR mosquito-borne |
