# Supplementary material for: Developing and validating modular surveys for vector-borne diseases: A study protocol
Source: PLoS One. 2025 Aug 25;20(8):e0323072. doi: 10.1371/journal.pone.0323072 (PMC12377612; doi:10.1371/journal.pone.0323072)
Supplement: Attachment — Submitted filename: Response to Reviewers.docx [file pone.0323072.s002.docx]

Dear Editor and Reviewers for PLOS ONE,

Thank you so much for providing the opportunity to submit a revised manuscript titled ***Developing and validating modular surveys for vector-borne diseases: a study protocol*** to PLOS ONE. We appreciate your insightful and valuable feedback on our manuscript. It substantially helped enrich the manuscript overall. We have incorporated your suggestions accordingly and highlighted changes within the manuscript using the track-change function. If you have any additional suggestions or concerns, please kindly let us know.

Reviewer #1: Comments on the protocol “Developing and validating modular surveys for vector-borne diseases: a study protocol”

1. Introduction
   a. The introduction highlights the increasing problem of VBDs in the U.S., but the specific issues this KAP study aims to address could be clearer. It would also be helpful to explain how modular surveys are unique and why they are better than traditional ones.
      i. **Response:** Thank you for your comment. We have addressed this in the introduction lines 83 – 95: It is increasingly recognized that standardized tools to collect data are needed to allow rigorous comparison across different contexts. These endeavors are still relatively nascent in vector-borne disease research. Ideally, KAP surveys use an underlying theoretical framework of behavioral theory, such as the Health Belief Model or the Risk, Attitudes, Norms, Abilities, and Self-Regulation framework, to help gauge individual perceptions of risk severity and self-efficacy. These underlying constructs are important to understand where efforts must be focused to enhance the adoption of prevention and control methods [6, 7]. The use of theoretical frameworks strengthens the rigor, focus, and relevance of the KAP research and captures important domains related to health action and behavior change. Standards across KAP surveys will enhance analysis and interpretation of the data across geographies and time. These studies set baseline values for subsequent evaluations, evaluate the efficacy of health education initiatives, and inform localized and culturally contextualized intervention approaches [8].
   b. While the manuscript mentions the HBM and RANAS frameworks, it doesn’t clearly explain how these concepts guide the design of the surveys.
      i. **Response:** As per your suggestion we have included the following statement in the introduction lines 90 – 93: The use of theoretical frameworks strengthens the rigor, focus, and relevance of the KAP research and captures important domains related to health action and behavior change. Standards across KAP surveys will enhance analysis and interpretation of the data across geographies and time.
2. Methods
   a. Workshop and Literature Review: The protocol mentions using a scoping review and a workshop to identify key topics for the survey. But it doesn’t say how they’ll pick the literature (e.g., inclusion/exclusion criteria) or how the important issues will be selected and ranked.
      i. **Response:** As per your suggestion, we further elaborated the inclusion criteria in lines 148 – 151.
   b. Delphi Process: CVR is a good way to check if survey items are relevant. However, the manuscript doesn’t explain what will happen if the experts don’t agree on certain items or how those disagreements will be resolved.
      i. **Response:** That's an excellent point. As discussed in our manuscript, CVR serves as a measure of agreement and disagreement among reviewers. According to the literature, items with a CVR value below 0.42 are excluded. To clarify this, we have provided additional explanation in lines 224 – 225.
   c. Cognitive Interviews: The manuscript suggests using cognitive interviews but doesn’t say how the feedback from these interviews will be studied.
      i. **Response:** We clarified that both qualitative and quantitative feedback will be obtained from the participants and survey items will be modified based on this feedback in lines 264 – 267.
   d. Survey Development: The manuscript identifies the general public as a key audience but does not address how demographic variability (e.g., language barriers, and literacy levels) will be managed in survey design.
      i. **Response:** More description was provided regarding the literacy level of the survey items (all survey items will be written at a 5^th^ grade level). We also clarified that the modules are not currently available in Spanish but the goal is to add this language in the future. Lines 285 – 288.
   e. Evaluation and Validation: The manuscript effectively outlines steps for content and face validity. However, more emphasis on reliability and external validity would strengthen the protocol.
      i. **Response:** Thank you for your suggestion. We already have measures in place to gauge reliability (Cronbach’s alpha) and ensure the modules have external validity through surveys targeting general populations. However, as per your suggestion, we included more details about that aspect in lines 274 – 284.
3. Discussion
   a. While the discussion highlights the potential for comparability and data uniformity, it would be beneficial to outline how public health agencies might implement these modular surveys or integrate findings into policy.
      i. **Response:** The outcomes of developing these modular surveys will depend on the teams that use them, but ideally there will be development and implementation of practical and sustainable interventions to reduce the burden of disease, which is explained in lines 377 – 398.
   b. A key limitation is the exclusion of Spanish-speaking populations from initial modules. While future plans are mentioned, the current protocol might miss critical insights from this demographic, especially in high-risk regions.
      i. **Response:** While we understand the critical insights that we may miss by not having the surveys available in Spanish, we do not have the time and resources to translate right now. A future goal is to undergo this validation process for other languages such as Spanish. Lines 287-288 and 394-396.
4. Line-specific Comments
   a. Line 119–120: Clarify what constitutes "sound measures" in the context of VBD surveys.
      i. **Response:** Removed the words “sound measures” and replaced with phrasing that is more understandable in line 144.
   b. Line 151–161: The integration of HBM and RANAS frameworks is insightful but briefly described. Include how these frameworks complement each other in survey item formulation.
      i. **Response:** We have included a description of the two frameworks in the methods section lines 186-196. The text is as follows: As the goal of KAP surveys for VBD prevention and control is to identify areas to focus efforts that motivate action, survey items will be mapped to the components of two theoretical frameworks for health behavior, the Health Belief Model (HBM) and the Risks, Attitudes, Norms, Abilities and Self-Regulation framework (RANAS) [6, 7]. Employing the HBM as a theoretical framework in survey development can be beneficial. This model helps gauge individual perceptions of risk severity as well as self-efficacy, or an individual’s belief in their ability to take meaningful action to mitigate risk [6]. Given that risk perception is intrinsically aligned with motivating behavior change, these elements are essential in gauging the likelihood of success of an intervention. The RANAS framework complements the HBM by addressing societal norms, which are not addressed in the HBM, thus emphasizing the importance of tailoring and contextualizing specific behaviors/behavior changes within a population [7].
   c. Line 183–188: The 4-point scale for CVR assessment is logical but could include a rationale for not using a broader scale (e.g., 5-point or 7-point Likert scale). **Sarah**
      i. **Response:** This decision is grounded in several key considerations from the literature, including the relationship between reliability and the number of response categories, the debate on the use of even versus odd category scales, and established guidelines for cutoff thresholds in the Delphi method. Based on these findings, we determined that including a neutral option (as in an odd-numbered scale) was not necessary. Instead, a four-point Likert scale was deemed sufficient for calculating the CVR value, as supported by previous research (Steiner at al., 2014, Lawshe, 1975).

d. Line 213–217: Include an explanation of how urban vs. rural insights will influence the modular survey design.
   i. **Response:** More description was provided regarding the insights that can be gained from urban vs. rural populations in lines 255-257.
e. Line 283–290: Discuss why Likert scales or time scales were inconsistently applied in previous studies and how this protocol standardizes these elements.
   i. **Response:** Researchers in past studies independently develop their survey tools. The selection of number of options and time scales was at their discretion, we do not have firsthand knowledge about why they made these choices. As stated in the protocol, our survey tools will develop items that researchers can use across multiple contexts. Using validated items will innately lead to consistency in the number of Likert options available.

5. General Comments
   a. Including a table summarizing the key domains and items identified in the literature review would enhance clarity.
      i. **Response:** As this is a protocol paper, we believed it may not be appropriate to include the preliminary findings including the domains and items. We will include the details for publication of our research once we collect and analyze the data.
   b. Check for minor typographical errors, such as repeated or fragmented phrases.
      i. **Response:** We reviewed the manuscript and made changes where necessary. If you have any additional issues, please let us know.
   c. If you wish to resubmit this study protocol once all ethics approval is in place, please ensure your methods section includes the following details: the study timeline for each phase including dates of recruitment, workshops, delphi surveys, data extraction for the literature review as well as the timeline for phase 3 interviews.
      i. **Response:** We have included an expected timeline table in lines 330-334.
