# Supplementary material for: Developing and validating modular surveys for vector-borne diseases: A study protocol
Source: PLoS One. 2025 Aug 25;20(8):e0323072. doi: 10.1371/journal.pone.0323072 (PMC12377612; doi:10.1371/journal.pone.0323072)
Supplement: Attachment — Submitted filename: Response to Reviewers .docx [file pone.0323072.s003.docx]

Dear Editor and Reviewers for PLOS ONE,

Thank you so much for providing the opportunity to submit a revised manuscript titled ***Developing and validating modular surveys for vector-borne diseases: a study protocol*** to PLOS ONE. We appreciate your insightful and valuable feedback on our manuscript. It substantially helped enrich the manuscript overall. We have incorporated your suggestions accordingly and highlighted changes within the manuscript using the track-change function. If you have any additional suggestions or concerns, please kindly let us know.

Reviewer #1: Comments on the protocol “Developing and validating modular surveys for vector-borne diseases: a study protocol”

1. There’s no mention of how missing data from cognitive interviews or surveys will be addressed.
   a. **Response:** This is an excellent point. We have included a statement in lines 251-257 describing how missing data from cognitive interviews will be handled, and a statement in lines 313-321 describing how missing data from surveys will be handled.
2. It’s unclear how many initial items per domain are expected to be tested, and how item reduction will be handled. Please add information, such as: "Initial item pools will intentionally include overlapping items within each domain to allow for assessment of internal consistency and clarity. Item reduction will be based.... and items that do not meet these criteria will be considered for removal or rewording.”
   a. **Response:** Thank you for the valuable feedback. Your concern has been addressed in lines 160-167.
3. There’s a well-described sampling plan, but no mention of how nonresponse bias will be handled. Add information on how you’ll assess or mitigate nonresponse bias, such as: “To assess potential nonresponse bias, we will compare basic demographic characteristics (e.g., age, gender, education level, residential area) between survey respondents and the broader invited sample, where such data are available. This will help identify systematic differences that may affect generalizability.”
   a. **Response:** Thank you for your comment. As per your suggestion, we have included a section in lines 300-304 outlining how non-response bias will be handled.
4. The discussion mentions that survey results can inform interventions but doesn’t explain how results will be used by agencies. Suggestion - Add a paragraph in the discussion proposing potential use cases, such as: tailoring educational campaigns, targeting high-risk communities, evaluating longitudinal behavior change following. interventions.
   a. **Response:** Thank you for your thoughtful comment and suggestion. We have added a description of how the results of these surveys can be used by different agencies such as public health, vector control, and applied research in our discussion section, lines 379-386.
